# Effect of Dead-Cell *Limosilactobacillus ingluviei* on Hematological Parameters and Jejunal Transcriptome Profile in Calves During the Weaning Period

**DOI:** 10.3390/ani15131905

**Published:** 2025-06-28

**Authors:** Chao Ban, Supreena Srisaikham, Xingzhou Tian, Pipat Lounglawan

**Affiliations:** 1 School of Animal Technology and Innovation, Institute of Agricultural Technology, Suranaree University of Technology, Nakhon Ratchasima 30000, Thailand; ban_chao@outlook.com; 2 Agricultural Innovation, Faculty of Agricultural Technology, Burapha University, Sa Kaeo Campus, Sa Kaeo 27160, Thailand; 3 College of Animal Science, Guizhou University, Guiyang 550025, China

**Keywords:** calf weaning, differentially expressed gene, jejunal epithelium, *Limosilactobacillus*, postbiotic, transcriptome

## Abstract

Weaning is challenging for dairy calves, frequently resulting in digestive issues. This highlights the importance of implementing appropriate nutritional strategies to enhance gut health and support optimal growth. Postbiotics is a promising alternative to traditional probiotics, conferring health benefits without the risks associated with live bacteria. This study aimed to investigate the effect of dietary supplementation with a postbiotic from dead-cell *Limosilactobacillus ingluviei* C37 (postbiotic LIC37) on blood biochemical parameters and jejunal epithelium transcriptomic profiles in calves. Fourteen Holstein bull calves were randomly allocated into two groups (n = 7). The control group (CON) received a basic diet, while the postbiotic group (DCLI) was supplemented with 1 g/d of postbiotic LIC37 for 90 days. Blood samples were collected on days 76, 83, and 90, respectively. The jejunal epithelial tissue was obtained from four randomly selected calves per group at day 90 for transcriptome analysis. The results showed that postbiotic LIC37 supplementation reduced globulin, total protein, neutrophil (Neu) levels, and neutrophil-to-lymphocyte ratio (NLR) levels in the DCLI group (*p* < 0.05). Transcriptomic analysis identified 76 differentially expressed genes (DEGs), with significant upregulation of genes involved in fatty acid metabolism (FABP1), intestinal barrier function (B4GALNT2), and detoxification (GSTA1), alongside downregulation of immune response regulation (FCRLA, FCRL4). Gene ontology (GO) and Kyoto Encyclopedia of Genes and Genomes (KEGG) analyses highlighted enrichment in pathways related to glutathione metabolism, drug metabolism, and vitamin digestion, indicating that postbiotic supplementation improved detoxification, oxidative stress defense, and nutrient absorption in calves. This study provides novel insights into the molecular mechanisms underlying the benefits of postbiotic LIC37 and supports its potential as a sustainable alternative to probiotics in calf nutrition.

## Simple Summary

Weaning is a stressful period for young calves, and it often leads to digestive and immune challenges. To help improve gut health during this transition, this study aimed to investigate the effects of a postbiotic from dead-cell *Limosilactobacillus ingluviei* C37 on the hematology and jejunal epithelial transcriptomes. The calves fed with this postbiotic showed lower levels of inflammation-related blood markers and beneficial changes in genes related to gut protection, nutrient utilization, and detoxification. These results suggest that postbiotics may help support the gut and immune health of calves during weaning, offering a safer alternative to traditional probiotics.

## 1. Introduction

Weaning is a particularly stressful and difficult event for dairy calves and can easily cause intestinal disorders, with a high prevalence primarily attributed to developmental factors during the early postnatal stages. Specifically, gut immaturity, weaker immune systems, and pathogen exposure primarily increase early-life intestinal disease risk [1]. Moreover, intestinal disorders not only compromise calf health, but also adversely affect weaning weights and subsequent milk yields [2]. Hence, ensuring intestinal health is key to maximizing both growth potential and production performance.

Dietary interventions could offer novel approaches to ameliorate inflammatory disorders by modulating the immune response through metabolic rewiring [3]. For decades, probiotics have been a staple in animal feed as additives to improve intestinal health [4]. Yet, the widespread presence of antibiotic resistance genes in these strains, and the demonstrated ability for these genes to transfer between organisms [5], casts a long shadow over their suitability for continued use as live bacteria in future nutritional strategies. Offering a compelling alternative to traditional probiotics, postbiotics are defined as a “preparation of inanimate microorganisms and/or their components that confers a health benefit on the host” [6], which could enhance host antioxidant capacity and immunity, regulate gut microbiota, and thereby support intestinal health. For example, Izuddin et al. found that feeding lambs with postbiotics from *Lactobacillus plantarum* RG14 could improve growth performance and nutrient intake [7]. Feng et al. demonstrated profound effects of postbiotics from *Bifidobacterium bifidum* B1628 on alleviating inflammation and intestinal damage in murine models [8].

RNA sequencing (RNA-Seq) is widely utilized to examine transcriptomic changes in key tissues in response to various intrinsic and extrinsic factors, offering insights into gene regulation and physiological adaptations [9]. Among segments of the gastrointestinal tract, the jejunum is particularly susceptible to stress [10]. However, most RNA-Seq studies in ruminant nutrition have focused on the rumen [11], colon [12], and liver [13], while studies investigating the jejunal epithelium in calves are relatively scarce. To fill this gap, we conducted RNA-seq on the jejunal epithelium of calves to identify DEGs and uncover the underlying molecular mechanisms associated with a postbiotic-supplemented diet. The findings of this study may broaden our understanding of how postbiotics affect intestinal health in calves and thus gain insight into the potential role of postbiotics in regulating calf health.

## 2. Materials and Methods

### 2.1. Animal Care

All animal procedures were conducted in accordance with the guidelines for the care and use of laboratory animals and were approved by the Animal Ethics Committee of Suranaree University of Technology (SUT-IACUC-0020/2023).

### 2.2. Treatments and Management

The postbiotic from dead-cell *Limosilactobacillus ingluviei* C37 was provided by the Laboratory of Monogastric Animal Nutrition and Feed Science at Suranaree University of Technology (SUT). The isolation and characterization of *Limosilactobacillus ingluviei* C37 were previously reported by Sirisopapong et al. [14]. Heat inactivation was performed at 80 °C for 30 min, following the method described by Tsukagoshi et al. [15].

All calves were obtained from a single farm. Within 3 h after birth, all calves were fed 2 L of colostrum via a nipple bottle, followed by an additional 2 L over the subsequent 12 h. At 24 h after birth, a hand-held refractometer (LH-Y12, Lohand Biological Co. Ltd., Hangzhou, China) was used to detect serum total protein. Calves with serum total protein concentrations greater than 5.6 mg/dL were selected for the experiment to ensure successful passive transfer of immunity [16]. Subsequently, calves were transitioned from colostrum to bucket-fed milk replacer (MR) starting at 3 days of age. They were transferred to the SUT farm, with the day of arrival considered as day 1 of the experiment.

A total of 14 Holstein bull calves (body weight, BW: 33.69 ± 5.28 kg, mean ± standard deviation), with ages of 5.71 ± 1.14 d, were randomly allocated to either a control group (CON, without postbiotic, n = 7) or a treatment group (DCLI, fed with 1 g/d of postbiotic LIC37 containing 10^8^ CFU/g, n = 7). The dosage was adapted from previous studies [17,18]. The calves were individually housed in 2.2 m × 2.4 m pens bedded with wood pellets over rubber mats. Soiled wood pellets were cleaned daily, with fresh pellets replaced weekly.

Milk replacer was purchased as a commercial product (Dairy-Rich Co. Ltd., Bangkok, Thailand). One kilogram of MR contained 974 g of dry matter, 88.7 g of ash, 226.7 g of crude protein, and 177.4 g of fat. It was fed at a concentration of 15% at 1.75% BW (air-dry basis) at 08:00 and 16:00. The feeding volume was adjusted weekly based on the BW. The postbiotic LIC37 was mixed with MR during the morning feeding, and the calves had free access to fresh drinking water. Beginning on day 33, the calves had free access to a commercial starter (973 calf starter, Charoen Pokphand Foods, Bangkok, Thailand). One kilogram of starter contained 909.4 g of dry matter, 89.7 g ash, 237.9 g of crude protein, 39.9 g of fat, 520.9 g of neutral detergent fiber, and 122.4 g of acid detergent fiber.

The feeding trial period lasted 90 days, and a weaning step-down protocol commenced on day 82, during which the MR allowance was reduced to 50% of the previous week’s volume. Calves were fully weaned by day 89. This abrupt reduction aimed to induce weaning stress for experimental evaluation [19].

### 2.3. Sampling Method

Blood samples were collected via jugular venipuncture by sterile tube without anticoagulation on day 76 (pre-weaning), 83 (mid-weaning), and 90 (post-weaning), prior to the morning feeding. They were subsequently submitted to the SUT hospital for the detection of hematological parameters, which encompassed total protein, globulin, albumin, and complete blood count.

Samples were collected at the end of the experimental period (d 90), prior to morning feeding. Four calves were randomly selected from each group and euthanized using captive bolt stunning and exsanguinated. The abdominal cavity was quickly opened. The jejunum was defined as starting at 100 cm caudal to the pylorus sphincter [20]. Jejunal samples were collected approximately 30 cm proximal to the collateral branch of the cranial mesenteric artery, rinsed three times with sterile phosphate-buffered saline (PBS, pH = 7.0), and immediately placed into sterile RNase-free tubes. The samples were then flash-frozen in liquid nitrogen and stored at −80 °C until RNA extraction.

### 2.4. RNA Extraction and RNA-Seq Library Construction

Total RNA was extracted from the jejunal epithelium using TRIzol reagent (Molecular Research Center, Cincinnati, OH, USA) according to the manufacturer’s instructions. The quality and quantity of the extracted RNA were analyzed using spectrophotometry (NanoDrop 2000 spectrophotometer; Thermo Fisher Scientific, Waltham, MA, USA) and 1% agarose (*w*/*v*) gel electrophoresis with a buffer of 0.5 × TAE. The RNA integrity numbers (RIN) were determined by capillary electrophoresis with a QIAxcel Connect using the QIAxcel ScreenGel Software (version 2.0); all the samples with RIN greater than 6.5 were used to construct the sequencing library.

RNA reverse transcription, library preparation, and RNA-seq were conducted at BGI Co., Ltd. (Shenzhen, China) using standard poly(A)-based enrichment and cDNA synthesis protocols. Libraries were sequenced on the DNBSEQ platform with PE500 read mode. Raw sequencing reads were quality-filtered using SOAPnuke (v1.5.6, RRID:SCR_015025) to remove adapter contamination, low-quality reads, and reads with >5% unknown bases.

### 2.5. Transcriptome Sequencing and Data Analysis

The resulting high-quality reads were retained as clean data and subsequently analyzed using the online multi-omics data mining system (biosys.bgi.com, accessed on 23 February 2025). The data were aligned to the reference *Bos taurus* genome (GeneBank Assembly ID: GCA_002263795.2) using HISAT2 version 2.2.1 [21] with default parameters, and gene-level quantification was performed with RSEM (v1.3.1) [22]. Differential expression analysis was conducted using DESeq2 (v1.4.5) [23], with DEGs defined as those with a fold change ≥ 1 and an adjusted *p* < 0.05. To identify enriched pathways, GO and KEGG enrichment analyses of DEGs were performed in R software (version 4.3.1) using the hypergeometric test, and terms with *p* < 0.05 were considered significantly enriched. The sequencing data have been submitted to the NCBI Gene Expression Omnibus (GEO) under accession number GSE293812.

### 2.6. Validation of DEGs by Quantitative Polymerase Chain Reaction

The RNA was transcribed into complementary DNA (cDNA) with the SweScript All-in-One RT SuperMix (G3337, Servicebio Technology Co., Ltd., Wuhan, China), following the manufacturer’s guidelines. Primers were designed using Primer3 software (https://primer3.ut.ee/, accessed on 23 February 2025) and synthesized by Servicebio Technology Co., Ltd. (Wuhan, China) (Table S1, https://doi.org/10.5281/zenodo.15302330, accessed on 23 February 2025). A total of seven DEGs in the same RNA samples were evaluated by quantitative polymerase chain reaction (qPCR) to verify the reliability and reproducibility of RNA-seq. These DEGs were Beta-1,4-N-Acetyl-Galactosaminyl transferase 2 (B4GALNT2), Fatty Acid-Binding Protein 1 (FABP1), Glutathione S-Transferase Alpha 1 (GSTA1), Paired Box 9 (PAX9), Paired Box 5 (PAX5), Fc Receptor-Like 4 (FCRL4), and Fc Receptor-Like A (FCRLA). The internal reference was Glyceraldehyde-3-phosphate dehydrogenase (GAPDH). The qPCR was carried out on the CFX Connect™ Real-Time PCR System (Bio-Rad, Hercules, CA, USA) using a 15 μL reaction mixture containing 7.5 μL of 2× Universal Blue SYBR Green qPCR Master Mix, 1.5 μL of each primer (10 μM), 2 μL of cDNA, and 4 μL of ddH_2_O. The thermal cycling protocol were: preincubation at 95 °C for 30 s, followed by 40 cycles at 95 °C for 15 s and 60 °C for 45 s (annealing and extension combined).

### 2.7. Statistical Analysis

Normal data distribution was confirmed using the Shapiro–Wilk procedure of SPSS (version 27, Chicago, IL, USA). The data on plasma biochemical parameters were analyzed using the General Linear Model (GLM) in SPSS to analyze variables that were repeatedly measured over time. Sphericity was assessed using Mauchly’s test, with the Greenhouse–Geisser correction applied whenever the assumption was breached. The significance level was set at a *p* value < 0.05.

## 3. Results

### 3.1. Effect of DCLI on Blood Metabolic Parameters in Calves

As shown in Table 1, no interaction between treatment and weaning was observed for any parameter. Postbiotic LIC37 supplementation decreases globulin and total protein levels (*p* < 0.05), without affecting albumin (*p* > 0.05). Additionally, postbiotic LIC37 supplementation significantly increased AGR levels (*p* = 0.027). Moreover, weaning had no effect on albumin and AGR levels (*p* > 0.05); but significantly affected globulin and total protein (TP) levels (*p* < 0.05).

### 3.2. Effect of DCLI on Complete Blood Count in Calves

As shown in Table 2, postbiotic LIC37 supplementation did not affect red blood cells (RBC), hemoglobin (HGB), and hematocrit (HCT) levels (*p* > 0.05). The weaning had no effect on most parameters (*p* > 0.05), but it significantly affected HGB levels (*p* = 0.036). Compared with the CON group, postbiotic LIC37 supplementation significantly decreased the levels of white blood cells (WBC), Neu, and NLR (*p* < 0.05), whereas lymphocyte (Lymp) levels remained unaffected (*p* = 0.126). Furthermore, neither weaning nor the interaction between treatment and weaning had any significant effect (*p* > 0.05) on WBC, Neu, Lymp, or NLR levels, respectively.

### 3.3. Quality of RNA-Seq Reads

A comparative RNA-seq analysis was performed to evaluate the effects of postbiotic supplementation on the transcriptomic profile of the jejunal epithelium in calves. The RNA-seq results for eight jejunal epithelium samples are presented in Table 3. The raw data reads ranged from 45.44 million to 47.19 million, with an average of 46.10 million. After filtering out low-quality reads, contamination, and other artifacts from the raw data, the number of clean reads ranged from 43.87 million to 45.15 million, with an average of 44.43 million. The GC content of clean reads varied from 49.26% to 52.10%, averaging 50.52%. Additionally, at least 95.70% of the reads had a sequence quality score greater than Q30 (percentage of bases with a Phred value ≥ 30). High-quality reads were mapped to the reference genome at a ratio of 97.11% to 98.04%, with an average of 97.53%.

### 3.4. DEGs Analysis

**Figure 1:**
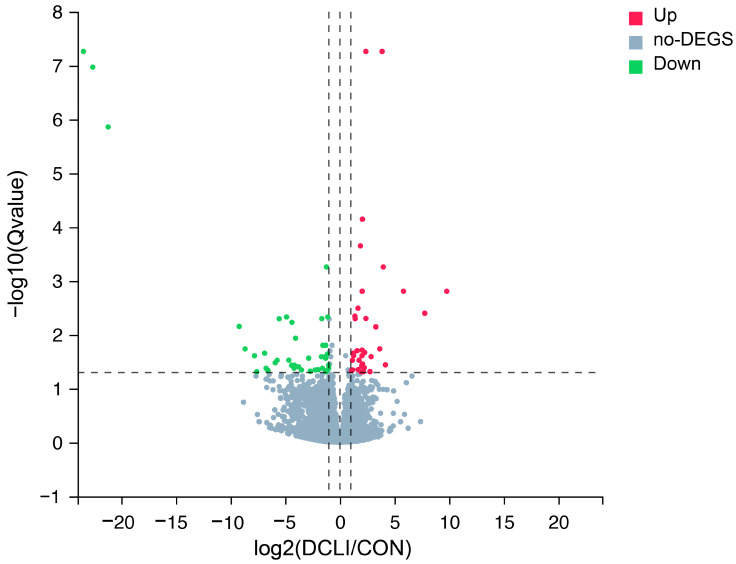
Volcano plot of differentially expressed genes in the jejunal tissue of calves. The genes meeting the conditions of adjusted *p* (Q value) < 0.05 and |log2 FC| ≥ 1 are considered as significant differentially expressed genes (DEGs), with red and green dots representing upregulated and downregulated transcripts, respectively. The vertical dashed lines represent the fold change thresholds, and the horizontal dashed line indicates the significance threshold. Gray dots represent insignificant DEGs. The x and y axes of the volcano plots show the log2 fold changes and –log10 q value, respectively.

Eight cDNA libraries were constructed to identify DEGs associated with the postbiotic response in the jejunum. As illustrated in Figure 1, a total of 76 DEGs were identified, comprising 36 upregulated and 40 downregulated genes. Detailed information on the DEGs is provided in Table S2 (https://doi.org/10.5281/zenodo.15302330, accessed on 29 April 2025). The genes related to metabolism and immunity were regulated by postbiotic supplementation, such as with Fatty Acid-Binding Protein 1 (FABP1) and Glucosylceramidase Beta 3 (GBA3), as well as by genes associated with intestinal barrier and immunity, such as Beta-1,4-N-Acetyl-Galactosaminyltransferase 2 (B4GALNT2), Glutathione S-Transferase A1 (GSTA1), and One Cut Homeobox 2 (ONECUT2). On the other hand, genes associated with inflammation, such as Paired Box 5 (PAX5), Paired Box 9 (PAX9), Fc Receptor-Like A (FCRLA), and Fc Receptor-Like 4 (FCRL4), showed downregulation, The top 20 upregulated and top 20 downregulated DEGs are presented in Table 4.

### 3.5. GO and KEGG Enrichment Analysis of DEGs

**Figure 2:**
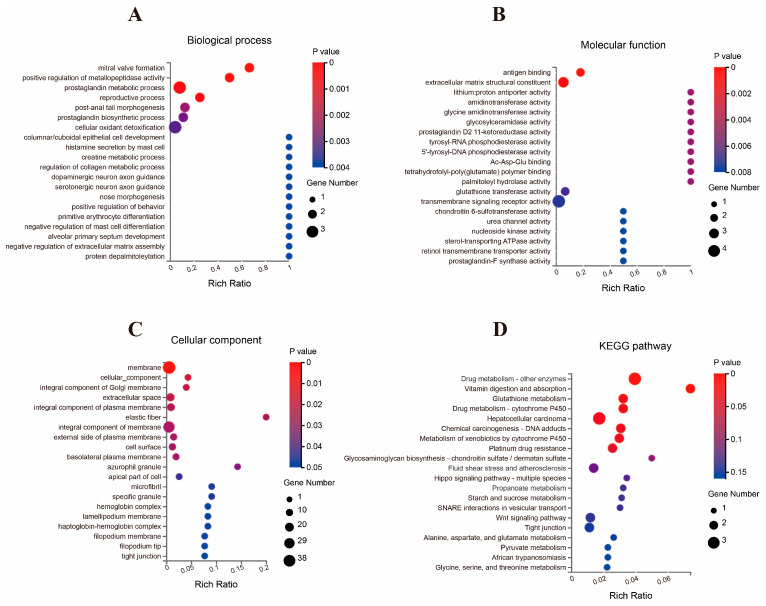
Top 20 enriched gene ontology terms of differentially expressed genes in the jejunal tissue of calves. (**A**) Biological process, (**B**) molecular function, (**C**) cellular component, (**D**) KEGG pathway. The circle size in each term corresponds to the number of genes. The circle’s color goes from blue to red, indicating a lower *p* value.

The GO and KEGG pathway analyses were performed on the 76 identified DEGs. The GO analysis classified these DEGs into three categories: biological processes (BP), molecular functions (MF), and cellular components (CC). A total of 352 enriched GO terms were identified, as detailed in Table S3 (https://doi.org/10.5281/zenodo.15302330, accessed on 29 April 2025). In the BP category, 152 GO terms were significantly enriched. The five most significant GO terms included mitral valve formation (*p* = 4.39 × 10^−5^), positive regulation of metallopeptidase activity (*p* = 8.76 × 10^−5^), prostaglandin metabolic process (*p* = 3.88 × 10^−4^), reproductive process (*p* = 4.05 × 10^−4^), and post-anal tail morphogenesis (*p* = 0.0017). For the MF category, 53 GO terms showed significantly enriched. Among these, the top five GO terms were transmembrane signaling receptor activity (*p* = 0.0065), actin filament binding (*p* = 0.0038), molecular function (*p* = 0.0200), extracellular matrix structural constituent (*p* = 0.0013), and peroxidase activity (*p* = 0.0084). In the CC category, 20 GO terms were significantly enriched. The top five most significant GO terms included membrane (*p* = 0.0031), cellular component (*p* = 0.0140), integral component of Golgi membrane (*p* = 0.0164), extracellular space (*p* = 0.0165), and integral component of plasma membrane (*p* = 0.0189). Figure 2A–C present the 20 most significantly enriched GO terms.

The KEGG pathway analysis of the jejunal epithelial tissue identified eight significantly enriched pathways, namely arachidonic acid metabolism (*p* = 0.0045), drug metabolism—other enzymes (*p* = 0.0020; bta00983), vitamin digestion and absorption (*p* = 0.0036; bta04977), glutathione metabolism (*p* = 0.0182; bta00480), drug metabolism—cytochrome P450 (*p* = 0.0182; bta00982), chemical carcinogenesis—DNA adducts (*p* = 0.0199; bta05204), metabolism of xenobiotics by cytochrome P450 (*p* = 0.0211; bta00980), hepatocellular carcinoma (*p* = 0.0198; bta05225), and platinum drug resistance (*p* = 0.0280; bta01524). Figure 2D presents the 20 most enriched KEGG pathways.

### 3.6. qPCR Validation of RNA-Seq

**Figure 3:**
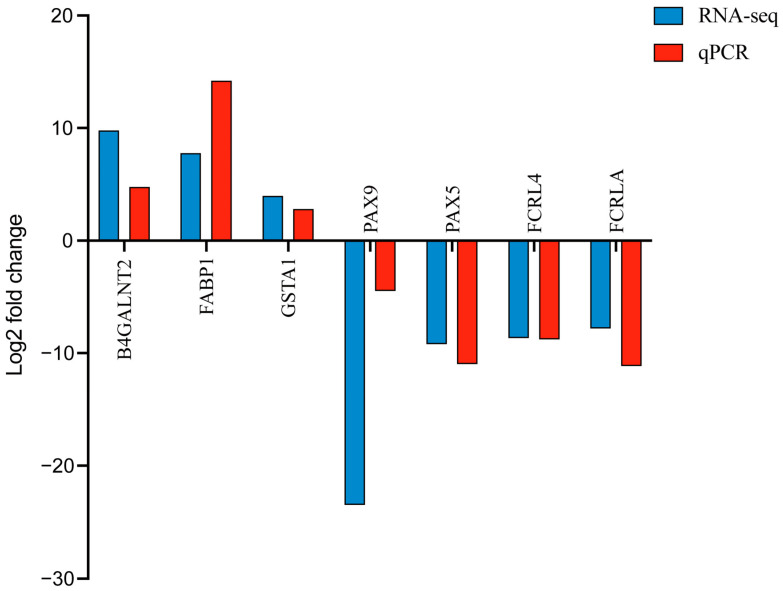
Quantitative PCR (qPCR) was employed to validate five DEGs in RNA-seq analysis. The *x*-axis denotes the genes, while the *y*-axis shows their mRNA expression levels as fold-change (FC) values. Expression levels obtained from RNA-seq and qPCR are illustrated by blue and red bars, respectively. B4GALNT2, Beta-1,4-N-Acetyl-Galactosaminyl transferase 2; FABP1, Fatty Acid-Binding Protein 1; GSTA1, Glutathione S-Transferase Alpha 1; PAX9, Paired Box 9; PAX5, Paired Box 5; FCRL4, Fc Receptor-Like 4; FCRLA, Fc Receptor-Like A.

To validate the RNA-seq results, we selected a subset of DEGs for qPCR analysis. Specifically, the expression levels of seven DEGs, including three upregulated genes (e.g., B4GALNT2, FABP1, and GSTA1) and four downregulated genes (e.g., PAX9, PAX5, FCRL4, and FCRLA), were quantified in the jejunal epithelial tissue. The qPCR results exhibited expression patterns consistent with the RNA-seq data, confirming the reliability and accuracy of our transcriptomic analysis (Figure 3).

## 4. Discussion

### 4.1. DCLI on Blood Parameters in Calves

Under stress conditions, pro-inflammatory factors such as IL-6, TNF-α, and IFN-γ typically increase [24], stimulating B cell proliferation, which in turn elevates globulin levels [25]. In addition, weaning stress increases intestinal permeability [26], leading to the entry of bacterial endotoxins (e.g., Lipopolysaccharide) into the blood and leading to a pro-inflammatory cascade [27], which increases the synthesis of acute phase proteins (APPs). In the present study, we observed elevated serum albumin and globulin levels in calves during weaning, which may be related to weaning stress [28]. Kim et al. reported that feeding calves antioxidants reduced total protein and globulin in the serum of calves during the summer growing period [29]. Therefore, we speculate that the lower levels of total protein and globulin, along with the higher AGR observed in the DCLI group, may indicate a reduction in the stress response due to postbiotic supplementation during weaning. This might due to the postbiotic enhancing intestinal barrier function [30] and reducing the penetration of harmful microorganisms and toxins [31], thereby reducing the degree of immune activation of the body. These findings align with previous studies. Wang et al. found that feeding neonatal calves with low-compound probiotics decreased total protein and globulin levels [32].

As the main component of red blood cells (RBCs), HGB directly participates in the transport of oxygen and carbon dioxide [33]. Weaning stress may activate the hypothalamic–pituitary–adrenal (HPA) axis, leading to increased cortisol production [34], which in turn may stimulate erythropoietin (EPO) to release additional HGB [35], thereby accelerating oxygen transport. On the other hand, weaning stress can cause intestinal disorders like diarrhea, leading to dehydration [36], which often alters HCT, HGB, TP, and electrolyte levels [37]. Consistent with changes in TP levels, weaning increased HGB, and a numerical increase in HCT values was also observed (*p* > 0.05), suggesting that there may be mild dehydration in calves. However, no obvious diarrhea was observed during weaning. Unfortunately, analyses of EPO and electrolytes were not included in this study, and we intend to investigate and validate them more comprehensively in subsequent studies. Consistent with our results, Ayyat et al. reported that feeding calves *Lactobacillus plantarum* did not affect HGB levels, whereas the weaning period led to an increase in HGB levels [38].

Neutrophils are key effector cells of the innate immune system [39], serving as the first line of defense against pathogens [40]. They respond to various signals by producing cytokines and inflammatory factors that regulate both inflammation and immune responses [41,42]. Weaning stress can activate the HPA axis in calves, leading to increased cortisol levels [34], which subsequently trigger a systemic stress response and an increase in neutrophil level [43]. In the present study, we observed a reduction in neutrophil count and NLR, along with stable lymphocyte levels, suggesting a potential attenuation of systemic inflammation and stress response in postbiotic-supplemented calves. This decline in neutrophil levels further echoes the previous decrease in globulin levels. This may be attributed to postbiotic improved gut barrier function and reduced microbial translocation, leading to decreased immune activation [44]. These findings align with the previous study of Izuddin et al. [45], who reported that post-weaning lambs fed postbiotics from *L. plantarum* RG14 exhibited significantly lower WBC and neutrophil levels, suggesting that postbiotics help modulate immune homeostasis by reducing unnecessary innate immune activation while maintaining adaptive immune functions.

### 4.2. DEGs in the Jejunum Epithelium

In ruminants, the rumen is not fully developed before weaning, making the small intestine the primary organ responsible for nutrient absorption and immune function [46]. A major function of the intestinal epithelium is to transport and present dietary and bacterial antigens to the immune system [47]. The transcriptome of the small intestinal epithelium may provide insights into key genes involved in the regulation of nutrient metabolism and immune function. Postbiotics contain cell wall components such as S-layer proteins and exopolysaccharides, which display antioxidant and antimicrobial properties [48,49]. Furthermore, they may aid in preventing pathogen adhesion to the gut [50], thereby acting as protectors of intestinal health. In the present study, we identified multiple genes associated with immune response and inflammation, metabolism and detoxification, and cellular signaling.

Among these, FABP1 was highly expressed in bovine jejunal epithelium and involved in fatty acid metabolism [51], which exerts a cytoprotective role by binding toxic molecules like free fatty acids and heme [52], while its antioxidant activity, driven by methionine and cysteine residues, helps reduce reactive oxygen species (ROS) production [53]. Previous studies have reported downregulated expression of FABP1 in the intestines of broilers under stress, such as heat stress [54] and high stocking density [55]. Consistent findings have been reported in monogastric animals. For instance, Wang et al. reported that feeding broilers with *Lactobacillus plantarum* 16 significantly upregulated mRNA expression of FABP1 in the ileal mucosa, which resulted in better transport and absorption of nutrients [56]. Similarly, an increased expression of FABP1 and CAT1 genes were observed in the ileal mucosa of broilers fed with *Bacillus subtilis* [55]. In the present study, we observed that the FABP1 gene was significantly upregulated in the DCLI group compared with the control group, suggesting that the postbiotic enhances FABP1 expression to bind toxic macromolecules while simultaneously inhibiting reactive oxygen species production, thereby providing comprehensive protection to intestinal epithelial cells.

Among the DEGs, B4GALNT2 is a glycosyltransferase responsible for synthesizing Sd(a)/Cad-antigen-like structures [57], which enhance intestinal barrier function, infection resistance, and immune homeostasis through glycosylation regulation, playing a crucial role in maintaining gut health [58]. In the present study, the expression of B4GALNT2 was significantly upregulated in the DCLI group, with a 7.44-fold change compared to the CON group. This upregulation was indicative of the postbiotic promoting the synthesis of numerous carbohydrate structures required for building Sd(a)/Cad-antigen-like structures, which confer protection to the jejunal epithelium. Similarly, Jiang et al. reported that feeding lactating cows with *Saccharomyces cerevisiae* fermentation product upregulated expression of B4GALNT2, thereby enhancing the ileum’s ability to defend against harmful molecules or microorganisms [59].

We observed that postbiotic LIC37 supplementation leads to an upregulation of GBA3 expression in the jejunal epithelium of calves. GBA3 is an enzyme with broad substrate specificity, capable of hydrolyzing various plant-derived β-glucosides, including phenolic glucosides, cyanogenic glucosides, isoflavones, and flavones [60]. The upregulation of GBA3 indicates an enhanced capacity of the intestine to process dietary plant glucosides [61]. These glucosides may be converted into more absorbable forms through hydrolysis and removal of their sugar moieties, thereby reducing their toxic effects on the host [62]. These findings indicate that postbiotic LIC37 supplementation may promote the absorption of these nutrients and reduce their potential toxic impact on the host by upregulating GBA3, which enhances intestinal detoxification and metabolic capacity for these dietary glucosides.

Glutathione S-Transferase A1 (GSTA1) exhibits GSH-dependent steroid isomerase activity as well as GSH-dependent selenium-independent peroxidase activity [63]. It has been demonstrated to protect cells from the detrimental effects of reactive oxygen species (ROS)-induced lipid peroxidation during oxidative stress caused by various factors [64]. In addition, GSTA1 also protects cells by binding to GSH and mitigating oxidative stress, thereby reducing subsequent lipid peroxidation [65]. Furthermore, a key function of GSTA1 is its role in inhibiting stress signaling kinases (JNK), which in turn influences the activation of caspases and the apoptosis cascade within the cell [66]. A previous study reported that treatment with antioxidants can reduced the apoptosis of porcine enterocytes by upregulating the expression of GSTA1 and regulating glutathione-related redox homeostasis [67]. Consistently, postbiotic LIC37 upregulated expression of GSTA1 in the jejunal epithelium of calves. This upregulation of GSTA1 may contribute to the protection of the intestinal epithelium by enhancing cellular antioxidant defenses and mitigating oxidative stress. Specifically, by increasing GSTA1 levels, postbiotic LIC37 supplementation may reduce oxidative damage, limit inflammation, and prevent cell apoptosis, ultimately supporting the integrity and function of the jejunal epithelium under stress conditions.

In the present study, we observed that postbiotic LIC37 supplementation enhances the expression of ONECUT2 in the jejunal epithelium of calves. This finding is consistent with previous research, indicating the crucial role of ONECUT2 in regulating cell proliferation, migration, adhesion, differentiation, and metabolism across various tissues such as the liver, pancreas, retina, neurons, and the immune system [68,69]. In particular, ONECUT2 has been shown to be essential for the development and differentiation of cells in these tissues. In ONECUT2 knockout mice, the lack of this gene led to failure to thrive during the critical period before weaning, with a 25–30% reduction in size and weight by postnatal day 19 and a higher mortality rate (only 70% survived before weaning) [70]. These findings highlight the importance of ONECUT2 in early development and suggest that postbiotic LIC37 supplementation may support the jejunal growth and differentiation of calves, potentially enhancing stress resistance during the weaning period. By upregulating ONECUT2, postbiotic LIC37 supplementation could contribute to maintaining intestinal barrier integrity, enhancing epithelial cell proliferation and differentiation, and reducing stress-related damage in the jejunum. Similarly, Jiang et al. reported that feeding lactating cows with a *Saccharomyces cerevisiae* fermentation product significantly upregulated the expression of ONECUT2 in the ileal epithelium [59].

On the other hand, the expressions of PAX5 and PAX9 were downregulated by postbiotic LIC37 supplementation. PAX5 and PAX9 are members belonging to the Pax gene family, which is involved in regulating various biological processes [71]. Specifically, PAX5 has been shown to inhibit several biological activities of B cells, including cell–cell communication, cell adhesion, cellular metabolism, migration, and nuclear processes [72]. The downregulation of PAX5 may increase PTEN expression and inhibit the PI3K-AKT signaling pathway, thereby reducing the secretion of TNF-α and IL-6 [73]. Regarding PAX9, while its role has been primarily studied in skeletal development [74,75], recent findings suggest a potential interaction between PAX9 and the NF-κB pathway [76], which is involved in oxidative stress, immune responses, and inflammation in the intestine [77]. Therefore, the downregulation of PAX5 and PAX9 by postbiotic LIC37 supplementation could further contribute to reducing inflammation and promoting intestinal health by modulating immune-related pathways.

We also observed that both FCRLA and FCRL4 genes were downregulated by postbiotic LIC37 supplementation, which may imply that postbiotic LIC37 supplementation could potentially help modulate immune responses by reducing the expression of these Fc receptor-like family members [78]. Given that FCRLA and FCRL4 are involved in the regulation of immune function and differentiation of B cells, the downregulation of these genes might be beneficial in attenuating inflammation and potentially promoting immune homeostasis [79,80]. Specifically, FCRL4 has been shown to act as a molecular switch in B cells, inhibiting adaptive immune signaling (such as BCR signaling) while enhancing innate immune signaling (e.g., TLR9 signaling) [80,81]. Therefore, postbiotic LIC37-induced downregulation of FCRLA and FCRL4 could contribute to a shift toward a more balanced immune response, potentially promoting a less pro-inflammatory environment and supporting intestinal health.

### 4.3. GO and KEGG Analysis

The GO analysis provides valuable insights into gene functions, elucidating key biological processes, molecular functions, and cellular components. In the present study, we identified several GO terms associated with intestinal barrier integrity and development, including columnar/cuboidal epithelial cell development, negative regulation of extracellular matrix assembly, regulation of collagen metabolic process, and tight junction. These findings suggest that postbiotic LIC37 supplementation may reduce immune activation induced by weaning stress. Additionally, GO terms related to antioxidant capacity, such as cellular oxidant detoxification, glutathione metabolic process, glutathione transferase activity, and peroxidase activity, indicate that postbiotic supplementation may enhance oxidative detoxification, thereby improving intestinal cell survival and reducing free radical damage. Furthermore, we identified GO terms associated with metabolic processes, including creatine metabolic process, creatine biosynthetic process, regulation of phosphate transport, and sterol-transporting ATPase activity, suggesting that postbiotic LIC37 supplementation may optimize nutrient absorption through these pathways.

In the present study, we used postbiotics to alleviate the inflammatory response in the intestinal tract of calves and speculated that it would significantly affect immune response in the jejunum. We found that the DEGs were significantly enriched in drug metabolism—other enzymes; vitamin digestion and absorption; glutathione metabolism; drug metabolism—cytochrome P450; hepatocellular carcinoma; chemical carcinogenesis—DNA adducts; and metabolism of xenobiotics by cytochrome P450. Among these, cytochrome P450-related pathways are mainly responsible for the biotransformation of exogenous compounds [82]. Glutathione (GSH) serves as a crucial antioxidant and detoxifying molecule, playing a key role in cellular defense, heavy metal chelation, and chemical detoxification [83]. Likewise, vitamins are essential for immune regulation, antioxidant protection, and energy metabolism [84]. Similar findings have been reported in previous research. For instance, in a transcriptome study of the ileal epithelium of lactating cows fed *Saccharomyces cerevisiae* fermentation product, significant enrichment was also observed in drug metabolism—other enzymes, glutathione metabolism, and drug metabolism—cytochrome P450 [59]. Similarly, Zhang et al. found that feeding weaned piglets with *Lactobacillus* led to significant enrichment in arachidonic acid metabolism, vitamin digestion and absorption, and metabolism of xenobiotics by cytochrome P450 [85]. Consistently, our study found that dietary supplementation with postbiotic LIC37 influenced similar pathways, suggesting that the postbiotic may modulate intestinal homeostasis by regulating detoxification, oxidative stress defense, and nutrient metabolism.

## 5. Conclusions

This study demonstrates that dietary supplementation with postbiotics from dead-cell *Lactobacillus ingluviei* C37 positively influence intestinal health in calves. Postbiotic supplementation reduced globulin and neutrophil levels, indicating its potential to mitigate systemic inflammation. Transcriptomic analysis revealed that the postbiotic modulates key pathways involved in nutrient metabolism and immune regulation by upregulating genes such as FABP1, B4GALNT2, and GSTA1, while downregulating pro-inflammatory genes like PAX5 and PAX9. These findings suggest that postbiotic LIC37 enhances intestinal barrier function and promotes immune homeostasis, thereby supporting calf health during the critical weaning period. Thus, postbiotic LIC37 represents a promising and sustainable feed additive with broad applications in ruminant nutrition.

## Figures and Tables

**Table 1:** Effect of DCLI on blood metabolic parameters in calves.

	Time ^1^									
	Day 76		Day 83		Day 90			*p* Value ^2^		
Item ^3^	CON	DCLI	CON	DCLI	CON	DCLI	SEM	Group	Time	G × T
Albumin, g/dL	2.79	2.87	2.91	2.97	3.14	3.34	0.069	0.433	0.078	0.299
Globulin, g/dL	3.31	2.76	3.61	3.01	3.87	3.37	0.087	0.008	0.006	0.596
AGR	0.85	1.04	0.81	0.99	0.82	1.01	0.029	0.027	0.091	0.921
Total protein, g/dL	6.10	5.53	6.53	5.63	7.01	6.71	0.117	0.027	0.002	0.252

^1^ Sampling time. ^2^ G × T, the interaction between group and time. ^3^ CON, control group (base diet); DCLI, base diet with 1 g of postbiotic (10^8^ CFU/g of postbiotic LIC37); SEM, standard error of the mean; AGR, the ratio of albumin to globulin.

**Table 2:** Effect of DCLI on complete blood count in calves.

	Time ^2^									
	Day 76		Day 83		Day 90			*p* Value ^3^		
Item ^1^	CON	DCLI	CON	DCLI	CON	DCLI	SEM	Group	Time	G × T
RBC, ×10^6^ cells/mm^3^	8.13	8.19	8.64	8.26	8.43	8.21	0.222	0.723	0.563	0.213
HGB, g/dL	7.77	7.89	9.07	9.26	8.74	8.90	0.230	0.740	0.036	0.930
HCT, %	24.43	25.71	27.43	28.57	27.57	27.36	0.612	0.555	0.137	0.248
WBC, ×10^3^ cells/mm^3^	10.23	7.70	11.29	8.50	12.44	9.77	0.385	0.006	0.155	0.938
Neu, ×10^3^ cells/mm^3^	3.31	1.95	4.40	2.59	5.11	3.10	0.243	0.003	0.192	0.411
Lymp, ×10^3^ cells/mm^3^	6.69	5.44	6.37	5.63	6.65	5.96	0.226	0.126	0.614	0.370
NLR	0.54	0.36	0.69	0.46	0.77	0.51	0.036	0.009	0.686	0.605

^1^ RBC, red blood cell count; HGB, hemoglobin; HCT, hematocrit; WBC, white blood cell count; Neu, neutrophils; Lymp, lymphocytes; NLR, the ratio of neutrophil to lymphocyte; CON, control group (base diet); DCLI, base diet with 1 g of postbiotic (10^8^ CFU/g of postbiotic LIC37); SEM, standard error of the mean; AGR, the ratio of albumin to globulin. ^2^ Sampling time. ^3^ G × T, the interaction between group and time.

**Table 3:** RNA-sequencing reads and mapping rates in the jejunal tissue in calves.

Sample Name	Raw Reads (Million)	Clean Reads (Million)	GC Content (%)	Clean Reads Q30 (%) ^1^	Total Mapping (%)
CON1	45.44	43.87	52.10	95.88	97.27
CON2	47.19	45.17	50.95	96.12	97.22
CON3	45.44	44.38	50.27	95.93	97.69
CON4	47.19	44.57	50.67	96.22	98.04
DCLI1	47.19	45.15	50.44	96.06	97.11
DCLI2	45.44	44.30	49.26	95.87	98.01
DCLI3	45.44	44.08	49.78	95.74	97.67
DCLI4	45.44	43.90	50.68	95.70	97.23
Average	46.10	44.43	50.52	95.94	97.53

^1^ Q30 indicates the percentage of bases with a Phred value ≥ 30.

**Table 4:** Top 20 upregulated and downregulated differentially expressed genes affected by DCLI in the jejunal tissue of calves.

Gene ID	Symbol ^1^	Log2 Fold Change	Q Value ^2^	Regulated
508108	B4GALNT2	9.7874	1.55 × 10^−3^	Up
327700	FABP1	7.7692	3.97 × 10^−3^	Up
539625	GBA3	5.8228	1.55 × 10^−3^	Up
786706	-	4.1697	3.61 × 10^−2^	Up
777644	GSTA1	3.9776	5.49 × 10^−4^	Up
539937	ARL14	3.8660	5.44 × 10^−8^	Up
782542	ONECUT2	3.6487	1.83 × 10^−2^	Up
511869	TM4SF5	3.2932	7.12 × 10^−3^	Up
505865	FOLH1B	2.8627	2.54 × 10^−2^	Up
538670	FAM151A	2.7599	4.83 × 10^−2^	Up
414732	GATM	2.3843	4.97 × 10^−3^	Up
525682	NOTUM	2.3823	5.44 × 10^−8^	Up
786760	BTN3A3	2.2747	2.13 × 10^−2^	Up
514667	MST1	2.2436	4.08 × 10^−2^	Up
100336768	ROS1	2.1404	4.85 × 10^−2^	Up
407225	MOGAT1	2.0725	3.49 × 10^−2^	Up
511097	SLC46A1	2.0696	7.13 × 10^−5^	Up
100300004	GLTPD2	2.0471	1.55 × 10^−3^	Up
282605	FAM13A	2.0424	1.92 × 10^−2^	Up
513137	TMEM72	2.0322	3.76 × 10^−2^	Up
540196	PAX9	−23.4653	5.44 × 10^−8^	Down
112444345	-	−22.6113	1.07 × 10^−7^	Down
112446673	-	−21.2060	1.37 × 10^−6^	Down
538371	PAX5	−9.2089	6.98 × 10^−3^	Down
534753	FCRL4	−8.6701	1.83 × 10^−2^	Down
782871	FCRLA	−7.8139	2.45 × 10^−2^	Down
531420	GP2	−7.6012	4.85 × 10^−2^	Down
504258	SIGLEC10	−6.8829	2.20 × 10^−2^	Down
407126	CR2	−6.7550	4.18 × 10^−2^	Down
107131854	-	−6.5830	4.60 × 10^−2^	Down
408008	KCNN1	−5.9122	3.30 × 10^−2^	Down
512439	HBA	−5.7393	2.97 × 10^−2^	Down
112445446	-	−5.5555	5.03 × 10^−3^	Down
493988	SLC14A1	−4.8759	4.67 × 10^−3^	Down
101908107	P2RY8	−4.6693	2.97 × 10^−2^	Down
515911	STRA6	−4.4179	3.68 × 10^−2^	Down
782922	-	−4.3880	5.88 × 10^−3^	Down
616320	SLC9B2	−4.1830	4.18 × 10^−2^	Down
100296105	-	−4.1574	3.68 × 10^−2^	Down
506550	TSPAN1	−4.0508	1.16 × 10^−2^	Down

^1^ B4GALNT2, Beta-1,4-N-Acetyl-Galactosaminyl transferase 2; FABP1, Fatty Acid-Binding Protein 1; GBA3, Glucosylceramidase Beta 3; GSTA1, Glutathione S-Transferase Alpha 1; ARL14, ADP-Ribosylation Factor-Like GTPase 14; ONECUT2, One Cut Homeobox 2; TM4SF5, Transmembrane 4 L Six Family Member 5; FOLH1B, Folate Hydrolase 1B; FAM151A, Family with Sequence Similarity 151 Member A; GATM, Glycine Amidino transferase; NOTUM, Notum, Palmitoleoyl-Protein Carboxylesterase; BTN3A3, butyrophilin subfamily 3 member A3; MST1, Macrophage-Stimulating 1; ROS1, Receptor Tyrosine Kinase 1; MOGAT1, Monoglyceride O-acyltransferase 1; SLC46A1, Solute Carrier Family 46 Member 1; GLTPD2, GLTP Domain Containing 2; FAM13A, Family with Sequence Similarity 13 Member A; TMEM72, Transmembrane Protein 72; PAX9, Paired Box 9; PAX5, Paired Box 5; FCRL4, Fc Receptor-Like 4; GP2, Glycoprotein 2; FCRLA, Fc Receptor-Like A; GP2, Glycoprotein 2; SIGLEC10, Sialic Acid Binding Ig-Like Lectin 10; CR2, Complement Receptor 2; KCNN1, Potassium Channel, Calcium Activated, Subfamily N, Member 1; HBA, Hemoglobin Subunit Alpha; SLC14A1, Solute Carrier Family 14 Member 1; P2RY8, Purinergic Receptor P2Y8; STRA6, Stimulated by Retinoic Acid 6; SLC9B2, Solute Carrier Family 9 Member B2; TSPAN1, Tetraspanin 1. ^2^ Q value is adjusted *p* value.

## Data Availability

The data presented in this study are openly available in the NCBI Gene Expression Omnibus (GEO) under accession number GSE293812. [BAN CHAO] [https://doi.org/10.5281/zenodo.15302330, accessed on 29 April 2025].

## Supplementary Materials

The following supporting information can be downloaded at https://www.mdpi.com/article/10.3390/ani15131905/s1, Table S1: List of primer sequences used for quantitative polymerase chain reaction (qPCR); Table S2: Differentially expressed genes (DEGs) in the jejunal tissue of calves; Table S3: Gene Ontology (GO) terms for the differentially expressed genes (DEGs) in the jejunal tissue of calves.
