# Supplementary material for: Effect of Dead-Cell Limosilactobacillus ingluviei on Hematological Parameters and Jejunal Transcriptome Profile in Calves During the Weaning Period
Source: Animals (Basel). 2025 Jun 28;15(13):1905. doi: 10.3390/ani15131905 (PMC12249185; doi:10.3390/ani15131905)
Supplement: Supplementary file 1 [file animals-15-01905-s001.zip › Table S1. List of primer sequences used for quantitative polymerase chain reaction (qPCR).pdf]

Table S1. List of primer sequences used for quantitative polymerase chain reaction (qPCR)

Accession number	Gene	Primer sequences (5' to 3')	Product size （bp）	Annealing temperature（°C）
508108	B4GALNT2	(F):TGA CCA ACT TCG CCA GAA CA	20	60
		(R):TTC CGC TCT GTT GAA ACG GT	20	60
327700	FABP1	(F):GTA CCA AGT CCA GAC CCA GG	20	60
		(R):GAT TTC CGA CAC CCC CTT GAT	21	60
777644	GSTA1	(F):GGA GAC AGA CTT TCG TAA GGA TTG A	25	60
		(R):ATC TTT TTC AGC AGG TGG GCA	21	60
540196	PAX9	(F):GTG GTC CCA ACA CTC CCT TT	20	60
		(R):CAT GAT TCA CCG AGA GCC CA	20	60
538371	PAX5	(F):CGA AGA ACA CGG ACA CCC TT	20	60
		(R):AAG AGC TTC TCG CCA TGT GA	20	60
534753	FCRL4	(F):CCA GAC ACT CGG CTT CAC TT	20	60
		(R):CAG TGT TTC AGC ACC ACA CG	20	60
782871	FCRLA	(F):AGC AGC CAC TGA GGA CAA TC	20	60
		(R):CTA ACA GTA TAC CAG GGG CAG T	22	60

B4GALNT2, Beta-1,4-N-Acetyl-Galactosaminyl transferase 2; FABP1, Fatty Acid Binding Protein 1; GSTA1, Glutathione S-Transferase Alpha 1; PAX9, Paired Box 9; PAX5, Paired Box 5; FCRL4, Fc Receptor Like 4; FCRLA, Fc Receptor Like A
